# Structural Change in Ni-Fe-Ga Magnetic Shape Memory Alloys after Severe Plastic Deformation

**DOI:** 10.3390/ma12121939

**Published:** 2019-06-17

**Authors:** Gheorghe Gurau, Carmela Gurau, Felicia Tolea, Vedamanickam Sampath

**Affiliations:** 1 Faculty of Engineering, ”Dunărea de Jos” University of Galati, Domnească Street, 47, RO-800008 Galati, Romania; gheorghe.gurau@ugal.ro; 2 National Institute of Materials Physics, POB MG-7, 77125 Bucharest-Magurele, Romania; felicia.tolea@infim.ro; 3 Department of Metallurgical and Materials Engineering, Indian Institute of Technology Madras, Chennai-600 036, India; vsampath@iitm.ac.in

**Keywords:** Ferromagnetic shape memory alloys, Ni-Fe-Ga, severe plastic deformation, SPD, HSHPT, ultrafine-grained materials

## Abstract

Severe plastic deformation (SPD) is widely considered to be the most efficient process in obtaining ultrafine-grained bulk materials. The aim of this study is to examine the effects of the SPD process on Ni-Fe-Ga ferromagnetic shape memory alloys (FSMA). High-speed high-pressure torsion (HSHPT) was applied in the as-cast state. The exerted key parameters of deformation are described. Microstructural changes, including morphology that were the result of processing, were investigated by optical and scanning electron microscopy. Energy-dispersive X-ray spectroscopy was used to study the two-phase microstructure of the alloys. The influence of deformation on microstructural features, such as martensitic plates, intragranular γ phase precipitates, and grain boundaries’ dependence of the extent of deformation is disclosed by transmission electron microscopy. Moreover, the work brings to light the influence of deformation on the characteristics of martensitic transformation (MT). Vickers hardness measurements were carried out on disks obtained by SPD so as to correlate the hardness with the microstructure. The method represents a feasible alternative to obtain ultrafine-grained bulk Ni-Fe-Ga alloys.

## 1. Introduction

Shape memory alloys (SMA) are functional materials which are predominantly used as actuators [1]. On the other hand, magnetic shape memory alloys (MSMA) may be used both as microactuators and displacement/force sensors or dampers [2,3]. In these alloys responsible for the shape memory effect is martensitic transformation (MT) in addition to the transition of magnetic order-disorder [4,5]. In fact, the shape memory effect and superelasticity underlie the fast response, reversible, and repeatable operation. Some of the most promising MSMA are Heusler-type ferromagnetic SMAs [6,7]. Heusler alloys are intermetallic compounds with the stoichiometry X_2_YZ, where X and Y represent transition metals and Z elements are from groups III, IV, or V [8]. Current industrial appliances are based on the reversible martensitic transformation between austenite (B2 or L21 structure) and martensite (tetragonal structure L10 unmodulated or modulated by seven or five atomic periods). MT has the effect of modifying the electronic structure and magneto-structural interactions (spin-phonon, electron-magnon). Thus the functionality of these alloys is linked. When the MT takes place at temperatures lower than the magnetic ordering temperature (Curie temperature, Tc), the alloy exhibits a ferromagnetic shape memory effect. Ni-Fe-Ga MSMA have close magnetic and martensitic transformation temperatures suitable for magnetic refrigeration applications [9]. Additionally, these alloy system have attracted attention by reason of their better ductility as compared with Ni-Mn-Ga alloys. Ni-Mn-Ga alloys are known to exhibit large magnetic field-induced strain and, thus, recommended as rapid magnetic actuators [5], but they are very brittle and difficult to shape [3]. Ni-Fe-Ga Heusler alloys are suitable for industrial applications avoiding brittleness, one of the major drawbacks of the Ni-Mn-Ga system [7]. The microstructure in this system is strongly dependent on the chemical composition and thermo-mechanical history since they influence both Tc and martensitic transformation temperatures [7,10]. For superior mechanical properties, including high strength and hardness together with increased fatigue resistance, originally coarse-grained alloys can be substantially refined down to nanometric structures [11]. Over the last two decades severe plastic deformation (SPD) has proven to be the most appropriate method to produce bulk ultrafine–grained and nanocrystalline materials [12,13]. Microstructural refinement by recourse of SPD is crucial for acquiring optimal mechanical properties in bulk Ni-Fe-Ga ferromagnetic SMA.

Our aim is to achieve severe plastic deformation in Ni-Fe-Ga MSMA applying a novel technique named high-speed, high-pressure torsion (HSHPT) that brings about grain refinement. This SPD technique is more suitable to process metallic alloys that are difficult to process. The present work investigates the properties of Ni-Fe-Ga with and without Co and Al substitution that were processed by HSHPT.

## 2. Experimental Procedure 

Buttons of Ni-Fe-Ga, Ni-Co-Fe-Ga, and Ni-Co-Fe-Ga-Al alloys were prepared by arc melting in argon using high-purity elements (99.99%) in appropriate proportion. The experimental procedure for preparing the bulk alloys and effect of substitutions on the structure and magnetic properties was described in detail earlier [7]. The bulk Ni-Fe-Ga-based alloy (with and without Co and Al substitution) was subjected to SPD so as to improve mechanical properties, as well as plasticity. The as-cast Ni_57_Fe_18_Ga_25_, Ni_50_Fe_22_Ga_25_Co_3_, Ni_52_Fe_20_Co_2_Ga_26_, and Ni_52_Fe_20_Co_3_Ga_23_Al_2_ in at% ingots (about 10 mm × 7 mm × 5 mm) were processed via HSHPT. We used a variation of the classical HPT technique whereby alloys were simultaneously subjected to compressive and torsion strain, described in detail in [12,13,14]. More specifically, in this process friction is introduced between the anvils and the sample by high speed rotation of the superior anvil. The chosen rotational speeds of the upper punch were 900 rpm and 1785 rpm, respectively. The deformation parameters were controlled through Programmable Logical Controller, PLC XC 200 (EATON, Germany). The degrees of deformation were calculated with Equation (1):

ɛ = ln(hi / hf),(1)

Bulk samples of all compositions were processed by the HSHPT technique with degrees of deformation varying between 0.81 and 2.82. The initial pressure applied from the bottom anvil was 10–50 bars. The compressive force recorded using Hottinger Spider 8 equipment was in the range of 0.0169–1.294 GPa. The achieved disks produced were up to 20 mm in diameter and 1.37 ± 0.08 mm in thickness. The entire process lasted 2–7 s. 

The microstructural investigation of Ni-Fe-Ga alloy was carried out on an OLIMPUS BX51microscope (Tokyo, Japan) equipped with a video camera and a QCapture software (QuickPHOTO MICRO 2.3). To enable a better scrutiny of morphology as well as the grain refinement introduced by HSHPT process a Zeiss (ZEISS EVO MA15, Munich, Germany) scanning electron microscope (SEM) was used. The severely deformed microstructure was also studied by transmission electron microscopy (TEM) (Model Tecnai 20G2, FEI, Hillsboro, OR, USA) operating at a voltage of 200 kV. In order to evaluate the elemental analysis, EDX investigations were carried out. Room temperature Vickers microhardness values were determined for the dual-phase characteristic of the alloys after processing by HSHPT.

The samples were subjected to thermal analysis by differential scanning (using a DSC Netzsch 204 F1 Calorimeter, Selb, Germany, with Proteus Software 2007) in the temperature range of 80–1000 K at a scanning rate of 20 K/min under a He atmosphere. This helped determine the characteristic transformation temperatures for the martensitic transformation. The samples were characterized for their magnetic properties (M (T)) in small and large magnetic fields, using a SQUID magnetometer (San Diego, CA, USA) in the RSO mode (for T <350K). 

## 3. Results and Discussion

### 3.1. Optical Microstructure Analysis

**Figure 1:**
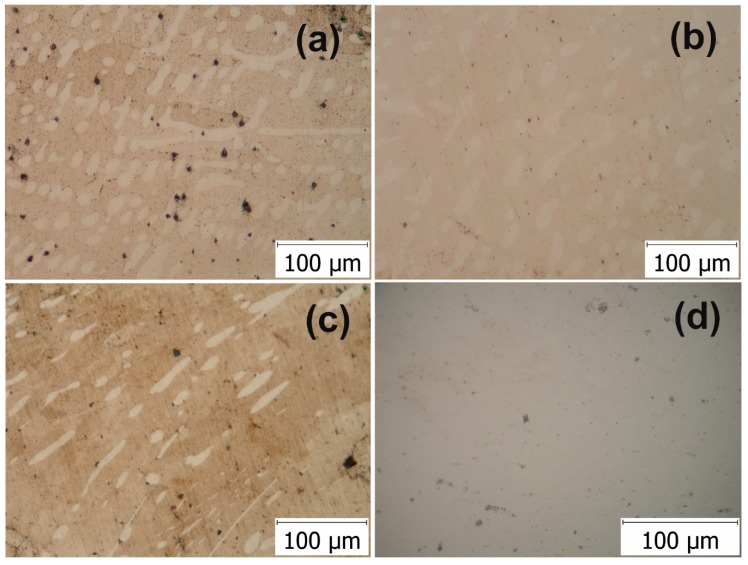
Optical micrographs of the HSHPT-processed disks of: (**a**) Ni_57_Fe_18_Ga_25_ alloy; (**b**) Ni_52_Fe_20_Co_2_Ga_26_ alloy; (**c**) Ni_50_Fe_22_Ga_25_Co_3_ alloy; and (**d**) Ni_52_Fe_20_Co_3_Ga_23_Al_2_ alloy.

Buttons of Ni_57_Fe_18_Ga_25_, Ni_50_Fe_22_Ga_25_Co_3_, Ni_52_Fe_20_Co_2_Ga_26_, and Ni_52_Fe_20_Co_3_Ga_23_Al_2_ in the as-cast state were successfully severely plastically deformed. After HSHPT, the disks present two phase zones in the microstructure, results that are in agreement with published work on the same system alloy deformed by hot-rolling [10]. It is well-known that in Ni-Fe-Ga-based alloys with Ga ≤ 27 at%, the composition has a typical β + ɤ two phase zone. In addition, the ɤ (FCC) second phase is considered responsible for the improved ductility of these alloys [7,10]. The effect of deformation of the matrix and phase precipitates is clearly manifested in the microstructure. Figure 1 illustrates the morphologies of severely plastically deformed Ni_57_Fe_18_Ga_25_-based alloy and the alloys with Co and Al substitution observed with the optical microscope. The micrographs of all alloys severely plastically deformed up to a logarithmic strain of 2 show quite similar dual-phase features: a matrix phase with a fine structure and an orderly network of second phase (Figure 1a–c). Such a dual-phase structure is consistent with that from an earlier work on Co-Ni-Ga alloy system [15]. The two phases were indicated as lamellar martensite and the ɤ second phase. Further TEM examinations showed that the microstructure of the severely deformed Ni-Fe-Ga alloy possessed a martensitic matrix.

The alloys under study manifest the apparent deformation more evident by increasing the level of deformation from 0.95 up to 2.52. The Ni_57_Fe_18_Ga_25_ specimen shows an orderly network of second phase expanded in radial and circular directions (Figure 1a). The ɤ consists of globular and elongated grains. In the case of the Ni_52_Fe_20_Co_2_Ga_26_ alloy that has undergone the same logarithmic strain of 0.95 as the base Ni-Fe-Ga alloy the ɤ grains have a globular morphology and a certain orientation along with shear direction (Figure 1b). The Ni_50_Fe_22_Ga_25_Co_3_ alloy shows elongated and fragmented second phase precipitates (Figure 1c). The curved morphology is associated with HPT processes. The technological application advantage of this alloys over the other MSMA is related to their improved ductility. This is actually linked to the low volume fraction of the secondary ɤ phase. The alloying with some additional elements (e.g., Co, Al) are suitable to promote the precipitation of ɤ phase [7]. In the alloy with Co and Al substitution that was processed to give rise to a logarithmic strain of 2.52 the individual ɤ grains or grain boundaries were not in a range detectable by standard optical microscopy observations (Figure 1d).

Combining high hydrostatic compression of the order of 1 GPa and high rotational speed (900 rpm or 1795 rpm) the HSHPT technology leads to large grain refinement in the studied alloys. The compressive force applied concomitant with torsion effort produces the refinement by grains shearing. Also the HSHPT processing technique combines a very efficient grain refining with the capability of keeping shape memory properties due to dynamic recrystallization as our research shows on other metallic alloys [12,13,14]. The technology leads to heat generation by intense friction between the anvils and the sample. The time span of processing (up to 7 s) control the prevalence of fine structure. The heat transferred by conduction from the sample to the tools helps achieve an ultrafine structure. In addition, the heat developed during processing by HSHPT method causes post deformation annealing (PDA) that is required after classical HPT to regain shape memory properties.

### 3.2. SEM Analysis

**Figure 2:**
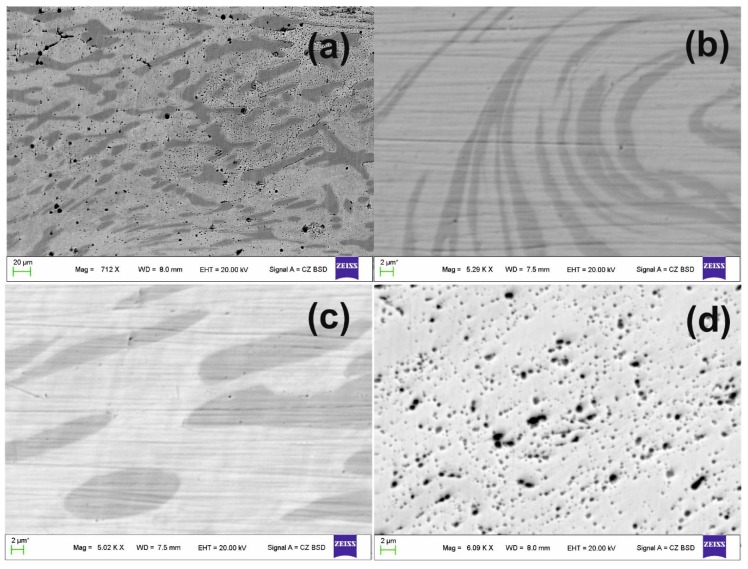
SEM micrographs of HSHPT processed Ni-Fe-Ga based MSMA subjected to different logarithmic degree of deformation: (**a**) 0.95 of the Ni_57_Fe_18_Ga_25_ alloy; (**b**) 1.45 of the Ni_52_Fe_20_Co_2_Ga_26_ alloy; (**c**) 0.81 of the Ni_50_Fe_22_Ga_25_Co_3_ alloy; and (**d**) 1.89 of the Ni_57_Fe_18_Ga_25_ alloy.

To understand the effect of the severe plastic deformation imparted by HSHPT on Ni-Fe-Ga magnetic SMA an investigation was performed by SEM-EDX. As expected, a significant fragmentation of ɤ -phase precipitates was observed in the severely plastically deformed microstructure (Figure 2). An important finding is the large refinement of martensite phase after severe plastic deformation by HSHPT. The dual-phase features (Figure 2a–c) may be noticed at low degree of deformation. It may be observed here that the deformed Ni_57_Fe_18_Ga_25_ sample (ɛ = 0.95) shows a matrix with no evidence of grain boundaries while the ɤ second phase is present. Increasing the level of deformation to ɛ = 1.45 in the Ni_52_Fe_20_Co_2_Ga_26_ sample leads to the generalized sliding path for ɤ -phase precipitates, as illustrated in Figure 2b. The Ni_50_Fe_22_Ga_25_Co_3_ alloy subjected to a low logarithmic strain level of 0.81 exhibited dual-phase features likewise the other three Ni-F-Ga alloy under study. The large grains of second phase were dispersed in the martensite matrix, as seen in Figure 2c. However, the increased degree of deformation (ɛ = 1.89) in the alloy without substitution reveals a highly deformed microstructure (Figure 2d). The feature pertaining to the martensite matrix could not be observed as they were outside the range of detection by SEM. Some precipitate particles were found while grain boundaries in the sample that were subjected to plastic deformation to the extent 1.89 could not be observed.

### 3.3. TEM Analysis

**Figure 3:**
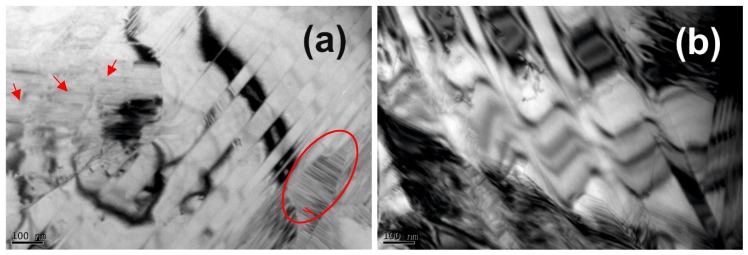
TEM bright field image of the Ni_57_Fe_18_Ga_25_ specimen after HSHPT: (**a**) Different features of martensite matrix: fine martensitic lamellae with inner microtwins indicated by the frame and very fine martensite plates indicated by arrows; and (**b**) the twinned structure of martensite matrix.

In order to study in detail the microstructural characteristics of the Ni_57_Fe_18_Ga_25_ magnetic SMA achieved by HSHPT processing, TEM images and were carried out. Bright field TEM image (Figure 3a) highlights different morphologies of martensite distorted by severe deformation. Some martensite variants are twinned and well self-accommodated.

Small grains of about 100 nm of fine martensitic lamellae are clearly identifiable on the right side of the micrograph. Inside ultrafine grains are nucleated high-density inner microtwins. The central area of the image shows serrated stress fields with a dark contrast. The stress field is typical of severe plastic deformation obtained via HSHPT [16]. Additionally, fine and ordered martensite lamellar plates can be observed on the left side of the image.

The twinned structure of martensite provide microstructural processes underlying the unique deformation propensity [17]. The MSMA alloys hold particular attribute of deformation recovery in addition to thermomechanical stimuli via applied magnetic field. The applied magnetic stimuli generate the driving force necessary for twin boundary movement. The differences between the martensitic variants (regarding magnetic domain) became very sensitive at applying magnetization field.

The zig-zag shaped of twin boundaries, which make-up martensitic variants of investigated alloy is highlighted in Figure 3b. It is known that ordered structure of the SMA lattice constitute one of significant factor for improved MSMA alloys.

### 3.4. EDX Analysis

**Figure 4:**
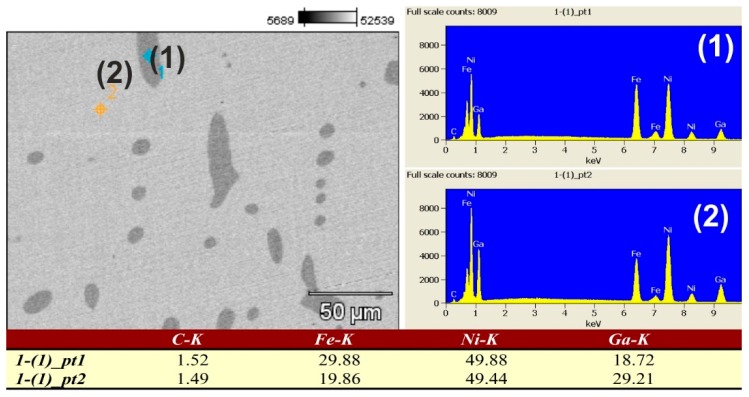
EDX elemental mapping for Ni_-_Fe_-_Ga alloy after severe plastic deformation: (1) second-phase (ɤ) and (2) martensitic matrix.

Figure 4 displays the composition profile of the Ni_57_Fe_18_Ga_25_ alloy surface. The EDX analysis results of different microstructural features are mentioned: point 1: second-phase (ɤ); and point 2: martensitic matrix. The martensitic matrix in this ternary alloy is found to be richer in Ga but leaner in Fe, as compared to the second-phase precipitates. The Ni content is about the same in all locations on the surface.

**Figure 5:**
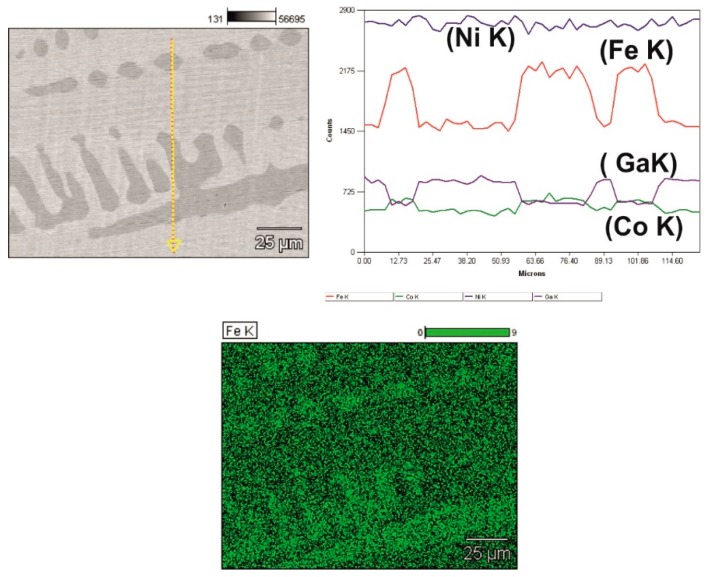
HSHPT’ed Ni_52_Fe_20_Co_2_Ga_26_ sample: EDX line across full thickness and EDX elemental mapping.

Figure 5 shows the variations in the Ni, Fe, Ga, and Co contents along the line across full thickness of severely plastically deformed Ni_52_Fe_20_Co_2_Ga_26_ disk. The corresponding EDX mapping analysis is illustrated, too.

The appearance of ɤ phase is accompanied by a sudden rise in the peak intensity corresponding to Fe and Co elements profile and drop in Ga content.

### 3.5. Microhardness Considerations

The results from the microhardness measurement are in good agreement with the microstructure of the studied alloys after processing by HSHPT. The hardness was enhanced with increasing the level of deformation. This is strictly correlated to the decrease in the size of the grains. The strengthening effect during plastic deformation is generated by the increase in the amount of grain boundaries which act as a strong obstacle for dislocation mobility through the material.

The phase-specific microhardness test reveals that martensitic matrix is harder (339 to 377 HV_0.02_) than the second phase (296 to 352 HV_0.02_) having relatively small differences between the Ni-Fe-Ga studied samples. Results were valid for all samples deformed at low degrees of deformation. The specimens severely plastically deformed to the extent of true strain of 2 showed a reversal of this trend. Unexpectedly, the ɤ phase became harder than the martensite. For example in the case of martensite in Ni_57_Fe_18_Ga_25_ MSMA the hardness value was 377 as against 383 for the ɤ phase.

### 3.6. Thermo-Magnetic Data

**Figure 6:**
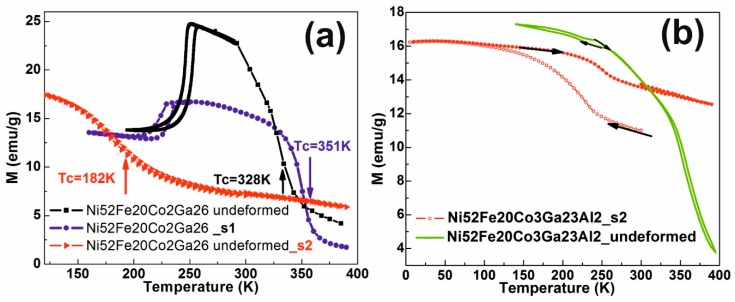
Variation of low-temperature magnetization variation (0.2 kOe) on: (**a**) Ni_52_Fe_20_Co_2_Ga_26_ undeformed and with two degrees of deformation (0.95 s1 and 2.2 s2) samples; and (**b**) Ni_52_Fe_20_Co_3_Ga_23_Al_2_ undeformed and with 2.2 degree of deformation.

Figure 6 presents significant results consisting of thermomagnetic curves for Ni_52_Fe_20_Co_2_Ga_26_ and Ni_52_Fe_20_Co_3_Ga_23_Al_2_ samples before and after severe deformation. Table 1 synthesized the characteristic MT temperatures and the magnetic ordering temperature (Tc) for the initial and severely deformed specimens.

As shown in Figure 6a, the deformed sample Ni_52_Fe_20_Co_2_Ga_26_ at 0.95 true strain presents the thermal hysteresis associated with reversible martensitic transformation.

The magnitude of the order-shift magnitude transitions and the way the structural transformations occur are reflected by magnetic properties. Martensitic transition is shifted to lower temperatures compared to those for the undeformed sample. The Curie temperature value increases to 351 K from 328 K for the undeformed sample. The magnetization of the deformed sample decreases. Marked changes occur in the severely deformed Ni_52_Fe_20_Co_2_Ga_26_ alloy at a very high degree of deformation: MT disappears, while the magnetic ordering temperature drops to 182 K (curve s1 in Figure 6a). This may be the result of the decrease in the size of the grains, reaching nanometric dimensions. The MT disappearing in the alloy calls for further investigation in the future. It can also be noted that the magnetization of all samples from Figure 6a does not drop to zero as the temperature increases, indicating the presence of a magnetic phase with a Tc temperature above 380 K. This is the ɤ phase which depletes the austenitic matrix in 3D-elements and is found in higher amount in the Ni_52_Fe_20_Co_2_Ga_26_ sample with ɛ = 2.2. The undeformed Ni_52_Fe_20_Co_3_Ga_23_Al_2_ sample (Figure 6b) describes a MT with a very narrow hysteresis. After a higher severe deformation the Ni_52_Fe_20_Co_3_Ga_23_Al_2_ alloy sample shows a very wide thermal hysteresis, which does not close after the specific cooling-heating MT cycle. The heat test does not reach the initial austenitic state, indicating unstable austenite.

## 4. Conclusions

The primary findings of the current study can be summarized as follows:

1. For the first time, buttons of Ni-Fe-Ga (with and without Co and Al substitution) in the as-cast condition were successfully severely plastically deformed by HSHPT at room temperature.
2. The microstructure of the two-phase Heusler Ni-Fe-Ga FSM alloys Ni_57_Fe_18_Ga_25_, Ni_50_Fe_22_Ga_25_Co_3_, Ni_52_Fe_20_Co_2_Ga_26_, and Ni_52_Fe_20_Co_3_Ga_23_Al_2_ after SPD was explored after SPD with an optical microscope, SEM-EDX, as well as TEM.
3. Martensitic transformation that takes place in severely deformed Ni-Fe-Ga alloys with Co and Al substitutions has been highlighted by magnetic measurements.
4. In the temperature range over which the martensitic transformation occurs a microstructural change take place producing discontinuities in the thermal dependence of magnetization.
5. The severe deformation at 0.95 logarithmic degree induces a decrease in MT temperatures, an increase in Tc and a decrease in magnetization, while a 2.2 degree of deformation induces a loss of the shape memory effect in the Ni_52_Fe_20_Co_2_Ga_26_ alloy, and unstable austenite in the Ni_52_Fe_20_Co_3_Ga_23_Al_2_ alloy.

## Figures and Tables

**Table 1:** The martensitic transformation temperatures and the magnetic ordering temperatures.

Sample.	Ms (K)	Mf (K)	As(K)	Af (K)	Tc (K)
Ni_52_Fe_20_Co_2_Ga_26_ _undeformed	243	218	250	258	328
Ni_52_Fe_20_Co_2_Ga_26__s1	224	207	230	238	351
Ni_52_Fe_20_Co_2_Ga_26__s2	-	-	-	-	182
Ni_52_Fe_20_Co_3_Ga_23_Al_2__undeformed	236	210	245	260	355
Ni_52_Fe_20_Co_3_Ga_23_Al_2__s2	240	145	238	267	>400
